# Construct validity and internal reliability of the healthcare provider performance scale

**DOI:** 10.3389/frhs.2026.1735784

**Published:** 2026-02-09

**Authors:** Khalid Alkhurayji, Saja Alrayes, Arwa Alumran, Abdallah Alsuhaimi

**Affiliations:** 1 Health Information Management and Technology Department, College of Public Health, Imam Abdulrahman bin Faisal University, Dammam, Saudi Arabia; 2 Executive Department of Standards, Saudi Central Board for Accreditation of Healthcare Institutions, Riyadh, Saudi Arabia

**Keywords:** health workforce, healthcare provider performance, instrument validation, occupational health, work performance, workplace

## Abstract

**Background:**

Assessing healthcare provider performance factors (HCPP) is crucial for enhancing the quality of healthcare services and the overall effectiveness of the healthcare system. Existing measurement tools often focus on limited aspects of factors independently and fail to comprehensively capture the organizational and individual factors influencing performance across diverse healthcare settings. To address this gap, this study developed and validated a multidimensional tool to assess HCPP across diverse settings.

**Methods:**

The validation process involved a two-phase analysis, in which Phase 1 used Exploratory Factor Analysis (EFA) to establish the initial factorial structure and Cronbach's alpha to assess preliminary internal consistency reliability, while Phase 2 employed Confirmatory Factor Analysis (CFA) to evaluate the model's fit, composite reliability, and construct convergent and discriminant validity.

**Results:**

EFA identified six factors consisting of Feedback and Organizational Support (FOS), Environment and Tools (ET), Incentives and Consequences (IC), Health Status (HS), Work-Family Conflict (WFC), and Healthcare Provider Performance (HCP),.explaining 63.7% of the total variance. The reliability of the scale was high (Cronbach's *α* = 0.837), with domains’ subscales ranging from 0.793 to 0.906. CFA confirmed the six-factor model with acceptable indices fit (CFI = 0.91, RMSEA = 0.08, GFI = 0.91). All factors loading exceeded 0.60 (*p* < 0.001). CR values ranged from 0.765 to 0.910, and AVE values supported the convergent validity (0.583–0.736). Discriminant for most of the constructs was established.

**Conclusion:**

The HCPP scale demonstrates acceptable psychometric properties, including acceptable reliability, factorial structure, and construct validity. The tool provides a robust measurement for assessing the factors associated with healthcare provider performance concerning individual and contextual-level determinants.

## 1 Introduction

Effective healthcare service delivery hinges on the performance of healthcare providers, whose roles are pivotal in ensuring service quality, patient safety, and system efficiency (1, 2). Many healthcare systems are becoming resource-constrained and complex-oriented (3, 4), with the growing demand to assess and evaluate HCPP given the inherently multidimensional factors influenced by organizational, individual, psychological, and contextual factors such as feedback systems, work environment, incentives and consequences, health status, and work-life balance (5–10).

The literature demonstrates substantial variations in the factors associated with HCPP across healthcare systems, countries, and contextual settings, with no unified domains or universal measurement scale reported. In fact, previous systematic reviews and meta-analyses reported multiple heterogeneous factors examined as isolated dimensions, such as incentives, health status, and organizational support, rather than a single construct. As a result, existing evidence remains fragmented, given that certain factors can be found in certain countries and not reported in other countries, with no validated multidimensional scale developed, which can comprehensively capture HCPP and the associated factors, particularly in Saudi Arabia. This lack of a contextually relevant, standardized measurement tool represents a global critical gap, limiting comparability across studies and the ability to inform practice and policy (11–13).

Despite the scales and instruments used to measure the factors associated with HCPP, which were factor-specific and were not designed to assess HCPP as an integrated, multidimensional construct, given that existing measurement tools often focus narrowly on clinical outcomes or task performance (14, 15), neglecting the importance of HCPP and the broader ecosystem in which healthcare providers operate, which encompasses work behaviors, individual competence, contextual, and organizational aspects that shape provider performance (16, 17). Along with this gap, none of the scales validated to capture organizational and individual-level factors within the context of the Saudi Arabia healthcare system comprehensively, which underscores the critical need for a psychometrically sound instrument that captures the full spectrum of the factors associated with HCPP (11–13). To illustrate the importance of this scale contextually, Saudi Arabia is undergoing a major health sector transformation under Vision 2030, in which the human resources for health are a key enabler for achieving the national vision (18). This national agenda aligns with the World Health Organization's Global Strategy on Human Resources for Health Workforce 2030, in which the first strategic objective is to optimize performance (19).From the perspective of the health sector and management, the impact of accurate measurement on performance lies in improving organizational effectiveness and healthcare service delivery. Additionally, performance measurement has been widely reported as tools used to support strategic alignment, operational decision-making, and workforce management (22, 23). Furthermore, population health management and healthcare services delivery depend on the HCPP, given that the associated factors can lead to a poor level of performance, which contributes to health inequities and poor quality of healthcare services (24, 25). Therefore, A valid and reliable tool can support management in general and the health sector in terms of performance benchmarking, informing workforce development strategies, and guiding interventions aimed at improving healthcare quality and service delivery (20, 21).

To address this need, the present study developed and validated a multidimensional scale to assess HCPP within diverse healthcare settings across Saudi Arabia. The instrument was designed to evaluate not only performance outcomes but also the organizational and individual-level determinants that shape provider behavior and effectiveness. Using a rigorous methodology that included Exploratory Factor Analysis (EFA), internal reliability, and Confirmatory Factor Analysis (CFA).

### 1.1 Instrument development

This instrument was developed based on previous study's framework identically (26, 27), given that it has been empirically tested across many countries. Followed by the execution of multiple focus group discussions among healthcare providers from various specialties, such as allied health professionals, physicians, and pharmacists, which resulted in the modification of HCPP dimensions after reaching thematic saturation with the recommended number of participants in each focus group (28). Furthermore, Fleiss’ kappa results in terms of the level of agreement across focus groups and co-researchers’ confirmation, reported 93.75% interrater rater reliability with free marginal kappa of the modified factors for the context of Saudi Arabia ]93%, Cl 0.78–1.00[. As a result, the final dimensions of the HCPP scale consist of healthcare provider performance (HCP). feedback and organization support (FOS), knowledge and skills (KS), clear job expectations (CJE), environment and tools (ET), incentives and consequences (IC), health status (HS), and work-family conflict (WFC). Subsequently, this study generated items based on the literature in preparation for the Delphi technique (29). The previous framework's items were used identically (26, 27), while other themes of the generated themes, such as WFC items, were extracted from a previous scale, given their weight in the literature and long-term empirical testing in many countries, which provide robust items to assess this domain (30). Additionally, HS domain items were extracted from a previous reputable scale that was used among many populations and contexts (31). Lastly, the HCP domain was adopted through generic items, given that these items were used across numerous industries and settings, including healthcare (32). In this scale, the scoring was based on a five-point Likert scale.

To ensure the applicability and appropriateness of content validation, two Delphi experts were consulted to evaluate the scales to build consensus on the Delphi method, following previous studies’ recommendations (33). Following the Delphi technique, a panel of experts comprising 20 healthcare professionals with extensive knowledge and experience in healthcare performance assessed the scale with three rounds. This iterative process continued until a consensus on the content and structure was reached. The Content Validity Index (CVI) was 0.89 for the scale. Furthermore, a pilot test was conducted with a small representative sample to improve item wording and/or remove ambiguity.

### 1.2 Dimensions of HCPP scale

The post internal consistency reliability and factor analysis, the HCPP scale consists of 39 items and aims to assess factors associated with healthcare provider performance [see Supplementary File S1]. This instrument score is based on a Likert scale from never to always.

#### 1.2.1 Feedback and organizational support

This domain, consisting of three items, aims to assess the performance-related interactions and organizational support experienced by healthcare providers. It includes statements designed to evaluate how often employees receive verbal and written reviews, as well as general feedback and completed feedback regarding their performance from their evaluators. Additionally, it examines the regularity of supervision within a six-month period, the extent to which employees are actively engaged in work by their organization, and whether the organization neglects their needs to perform at their best. Furthermore, it addresses the provision of employee orientation programs. Respondents are asked to indicate how frequently these events occur using a five-point Likert scale: Always, Often, Sometimes, Rarely, or Never, selecting the most appropriate response for each statement. The construct shows good internal consistency among the healthcare providers (Cronbach's *α* = 0.793).

#### 1.2.2 Environment and tools

This domain consisted of four items assessing the statements in terms of how frequently employees perceive their workplace as adequate, and whether they have access to the necessary equipment, instruments, and supplies required to perform their duties effectively. It also examines the level of satisfaction with work regulations and how often employees experience a positive organizational culture. The construct shows good internal consistency among the healthcare providers (Cronbach's *α* = .906).

#### 1.2.3 Incentives and consequences

This domain consists of two items that aim to assess the frequency and types of rewards or penalties experienced by employees in relation to their performance. This section explores how often employees receive non-monetary incentives from their employer, as well as how often they face disincentives for poor performance. The construct shows good internal consistency among the healthcare providers (Cronbach's *α* = 0.838).

#### 1.2.4 Health status

This domain consisted of three items designed to evaluate the overall physical and emotional well-being of employees. The statements explore the frequency of health-related limitations and emotional states. These include how often respondents experience limitations in daily activities, as well as how frequently they encounter pain or face restrictions in social activities. The construct shows good internal consistency among the healthcare providers (Cronbach's *α* = 0.800).

#### 1.2.5 Work-family conflict

This domain consisted of two items that focus on understanding the extent to which family responsibilities impact employees’ professional lives. The statements in this section assess how often family demands interfere with work-related activities, and the degree to which family matters affect performance. The construct shows good internal consistency among the healthcare providers (Cronbach's *α* = 0.792).

#### 1.2.6 Healthcare provider performance

This domain consists of three items, the statements of which explore how frequently individuals were able to plan and complete their work on time, remain focused on desired outcomes, and set priorities effectively. The section also assesses the ability to manage time well and initiate new tasks independently once previous tasks have been completed. The construct shows good internal consistency among the healthcare providers (Cronbach's *α* = 0.801).

#### 1.2.7 Other dimensions of HCPP

Given that the initial EFA indicated a six-factor structure, the domains *Knowledge and Skills* and *Clear Job Expectations* were initially included in the analysis. However, several items under these two domains showed weak factor loadings or substantial cross-loadings, failing to meet the minimum threshold for retention. As a result, these domains were excluded during EFA refinement process. Subsequent CFA further demonstrated that the removal of these two domains substantially improved the model's overall fit and psychometric properties. The decision to exclude *Knowledge and Skills* and *Clear Job Expectations* was also supported conceptually. The original measurement framework was adapted by adding new domains and applying it to a different population within a distinct geographic and cultural context. This necessitated a re-evaluation of the tool's structural validity to ensure contextual relevance and conceptual alignment (26, 27). Therefore, EFA was employed to empirically assess how items grouped together in this new setting, ensuring that the adapted instrument retained both construct validity and internal consistency, which was subsequently confirmed by CFA.

## 2 Materials and methods

This study used a cross-sectional design with a multi-stage stratified cluster sampling technique with proportional allocation, where the population was clustered across the 13 administrative regions, followed by stratification of healthcare providers Healthcare within each region by professional category ensure fair representation of Saudi Arabia, based on the distribution of healthcare providers reported in the Statistics Book of the Ministry of Health (34). The initial estimated sample size was 384, calculated using the standard formula from a total healthcare provider population of 264,327, through an online website (35), assuming a 95% confidence, Margin of Error of 0.05, and a population proportion of 0.5 (36). To account for the complexity introduced by stratification, a design effect (multiply by 300%) was applied, increasing the sample size to 1,152 (37). This adjustment addresses intra-stratum homogeneity and enhances statistical power across regions and specialties (38). Proportional stratification ensures that responses are not dominated by highly populated regions such as Riyadh and Makkah while still capturing data from smaller regions such as Albaha and the Northern Borders. Furthermore, the sample was rounded up to 1,153 to guarantee that at least one participant was included from each administrative region and each relevant stratum, including key specialties such as physicians, allied health professionals, and pharmacists. This approach also supports comparative analysis between different clinical roles, ensuring the sample reflects the full spectrum of healthcare provider experiences across Saudi Arabian regions. The following formula is used to determine the proportional sample size allocated to each region in a stratified sampling design, based on the total population of healthcare providers across all regions (39, 40).

$$\left(Sample\;Size\;for\;Region_{i}=(Population\;of\;\;Region_{i}/Total\;Population)\times Total\;Sample\;Size\right)$$

Where:

Population of Region_i_ = Number of healthcare providers in each region.
Total Population = 264,327 (Total number of healthcare providers across all 13 regions).
Total Sample Size = 1,152 (after applying a design effect of 3).

### 2.1 Participant

Analysis of many variables, such as CFA, is generally too complex to be prospectively powerful. Little is known about the observed and latent variables’ clinical differences and standard deviations (41). Despite this, we used the sample size approach (two independent subsamples randomly drawn from the full dataset, used one for EFA and the other for CFA) as recommended in the literature (42, 43), that involves 100 sample size = fair, 200 sample size = good, 500 sample size = very good, and > 1,000 = excellent (44).

### 2.2 *A priori* model

Since the original framework was modified by adding two domains and applied to a different population in a new geographic and cultural context, it was necessary to re-evaluate the underlying structure of the measurement tool. Therefore, EFA was conducted to empirically assess how items grouped together in this new setting. This step ensured that the modified instrument retained conceptual clarity and construct validity. Nonetheless, the data were evaluated using the Measure of Sampling Adequacy Kaiser-Meyer-Olkin (KMO) Measure and Bartlett's Test of Sphericity. Following EFA, a CFA was performed to test the stability and fit of the emergent factor structure. The use of both EFA and CFA is a standard and robust approach when validating adapted instruments in new contexts (45). CFA was executed through varimax factor analysis to determine the nature and number of factors in the instrument being assessed. Six factors were produced from this analysis. This model was chosen based on comparing the varimax with principal axis and oblique rotation (46). The latter of these models was more realistic and revealed a correlation among these factors.

### 2.3 Statistical analysis

The validation was executed using confirmatory factor analysis in AMOS Version 26.0 (47). The result suggested that 6 factors should be included in the final CFA model. The fitting of CFA model used maximum likelihood estimation. Given that the raw and modified ×^2^ are usually upwardly biased in terms of sample size (48). To test the impact of high sample size, we randomly selected and reduced the sample size, followed by the execution of a *post hoc* analysis, resulting in new raw and scaled *χ*2 values of 1,093. The *χ*2 reduced significantly with the reduction of sample size, which harmonizes with previous and other studies (49). Therefore, alternative indices were estimated; GFI greater than 0.9 was considered a good fit (50). The Root Mean Square Error of Approximation (RMSEA) evaluates the model fit by assessing the parameters’ fit towards the population Covariance matrix (51), and RMSEA value of less than 0.08, suggesting acceptable fit (52). The internal consistency of the HCPP scale was assessed using Cronbach's alpha. To evaluate Convergent validity, the Average Variance Extracted (AVE) for each construct was evaluated against its correlation with the other constructs. Where AVE was larger than the construct's correlation with other constructs, then Convergent validity was considered to be confirmed (46). Discriminant validity was established where Maximum Shared Variance (MSV) and Average Shared Squared Variance (ASV) were both lower than Average Variance Extracted (AVE) for all the constructs (53). The total score for each subscale was computed using the loadings for each item produced from CFA (54).

## 3 Results

The majority of participants were Saudi nationals, constituting 71% of the sample, while 29% were non-Saudis. In terms of marital status, 56.8% of the respondents were single, 42.6% were married, and only 0.6% were divorced. Regarding gender, the distribution was relatively balanced, with 52.1% being male and 47.9% female. The participants’ clinical education levels were primarily at the bachelor's level (61.5%), followed by post-graduate degrees (36.3%), and a smaller proportion held diplomas (2.2%). In terms of specialty, a significant portion of the sample, 73.5%, were allied health professionals, while 24.5% were physicians, and 2% were pharmacists. As for the hospital level in which the participants worked, the majority, 56.8%, were affiliated with tertiary-level hospitals, followed by primary-level (28.4%) and secondary-level hospitals (14.8%).

The highest proportions of participants came from the Makkah region (19.7%) and Riyadh (19.4%), together accounting for nearly 39.1% of the total sample. These were followed by the Eastern Region, contributing 15.1% of respondents. Other regions with notable participation include Madinah (7.5%), Aseer (7.3%), and Qassim (6.6%), while Jizan and Najran contributed 5.7% and 3.5%, respectively. Smaller percentages were observed from Tabouk (3.6%), Aljawf (3.7%), Hail (3.4%), and the Northern Borders (2.4%). The region with the lowest representation was Albaha, comprising 2.2% of the sample.

### 3.1 Exploratory factor analysis

The analysis of data showed that KMO value was 0.807, indicating meritorious sampling adequacy as well as Bartlett's test, which was statistically significant [*χ*²(276) = 17,557.213, *p* < .001], confirming that the correlations across items were sufficient for factor analysis. Table 1 shows that by using Maximum likelihood extraction method with varimax rotation, six factors were extracted based on eigenvalues greater than 1. Collectively, the six factors accounted for a cumulative total variance of 63.70%. Furthermore, the matrix of rotated factors revealed a clean structure across factors with primary loadings above 0.50 and minimal cross-loadings with minimal cross loadings, allowing for a clear interpretation of factors.

**Table 1:** Results of the exploratory factor analysis of the HCPP scale.

Factor	Environment and tools	Feedback and organizational support	Incentives and consequences	Health status	Work-family conflict	Healthcare provider performance
ET1	0.747					
ET2	0.844					
ET3	0.923					
ET4	0.855					
ET5	0.703					
ET6	0.702					
FOS2		0.622				
FOS5		0.820				
FOS6		0.692				
IC2			0.804			
IC3			0.660			
HS2				0.713		
HS4				0.715		
HS5				0.610		
WFC1					0.751	
WFC2					0.712	
WFC3					0.872	
HCP2						0.698
HCP3						0.746
HCP4						0.803
HCP5						0.757
HCP7						0.626
HCP9						0.565
Eigenvalue	1.568	1.002	2.961	1.137	6.460	3.594
Explained variance	7.858	5.958	9.678	7.396	19.068	13.745
Total explained variance	63.703					

HCP, healthcare provider performance; FOS, feedback and organization support; ET, environment and tools; IC, incentives and consequences; HS, health status; WFC, work-family conflict.

In terms of each factor, items related to Environment and tools (ET1-ET6) reportedstrong loading, ranging from 0.71–0.92. Factor 2 consists of items measuring healthcare provider performance (HCP2–HCP9), with factor loadings ranging from 0.57 to 0.79. Regarding factor 3, which comprised items related to work–family conflict (WFC1–WFC3), which showed that all factor loadings were above 0.71. In terms of factor 4, which reflected feedback and organizational support, including (FOS2, FOS5, and FOS6) that illustrated factor loading ranging from (0.61–0.80). Furthermore, Factor 5 consisting with Health Status items (HS2, HS4, HS5) showing factor loadings between 0.611 and 0.732. Lastly, Factor 6 reflected the incentives and consequences domain, with only two items (IC2–IC3) loading between 0.858 and 0.617.

**Figure 1:**
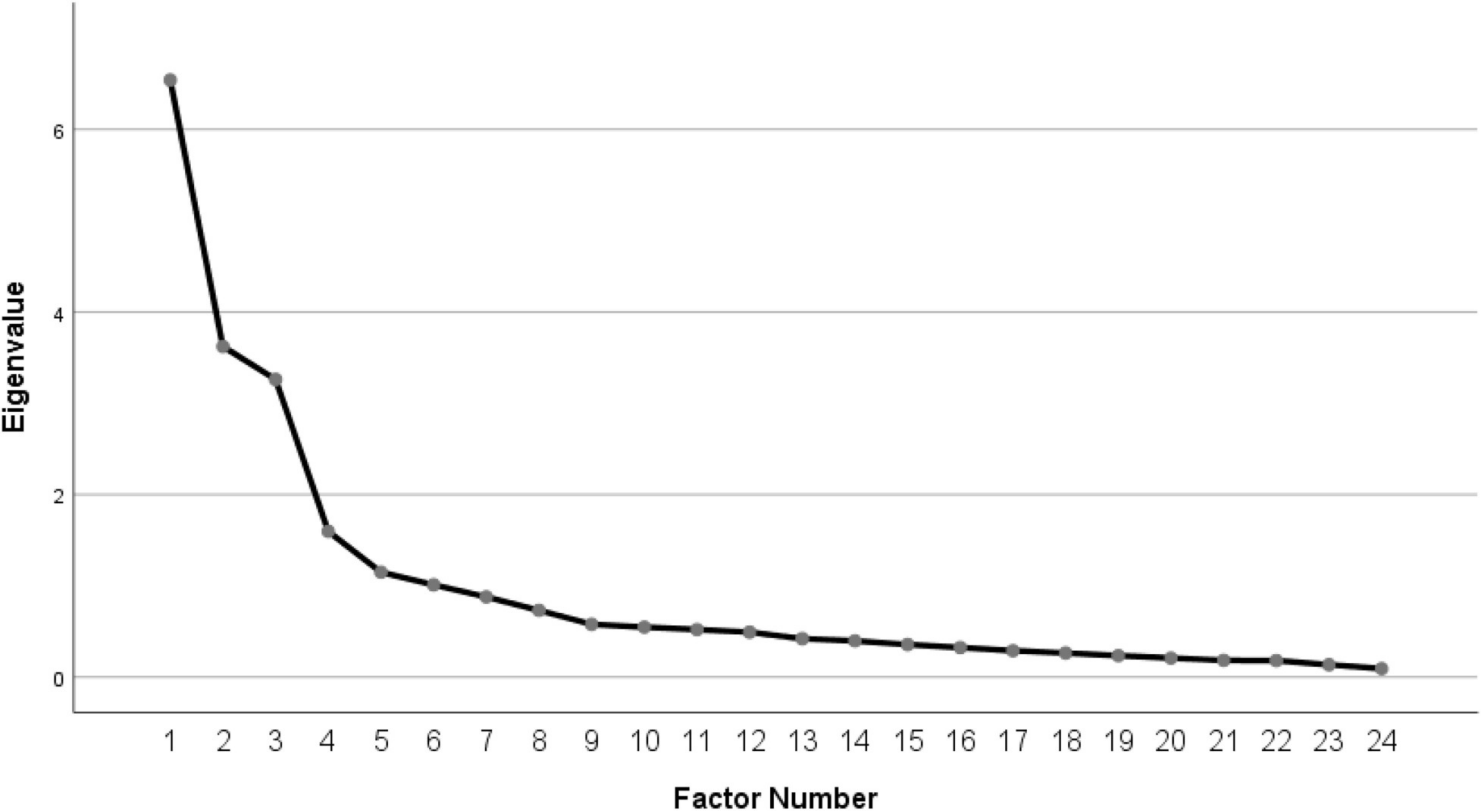
Scree plots of exploratory factor analysis.

As illustrated in Figure 1, the scree plot shows a steep decline in eigenvalues from factor 1 to factor 3, with the curve beginning to level off gradually starting at factor 4. The clear inflection point observed before factor 7 supported the decision to retain six factors.

### 3.2 Internal reliability

Overall instrument reliability (Cronbach's *α* = 0.837) demonstrated excellent internal consistency across the scale and subscales. The Environment and Tools domain has the highest level of reliability (*α* = 0.906), followed by Feedback and Organizational Support (*α* = 0.793) as well as incentives and consequences (*α* = 0.838). Furthermore, the Health Status domain (*α* = 0.800), work-family Conflict (*α* = 0.792), and the Healthcare Provider Performance subscale (*α* = 0.801) also have a high level of reliability (Table 2).

**Table 2:** Descriptive statistics of the HCPP internal consistency scale.

Items	Item total correlation	Cronbach's α if item deleted	X ± SD
Environment and tools		0.906	3.79 ± 0.92
ET2 How often do you have the necessary equipment to perform your job?	0.895	0.87	2.81 ± 1.0
ET3 How often do you have the necessary instruments to perform your job?	0.932	0.848	2.90 ± 0.986
ET4 How often do you have the necessary supplies to perform your job?	0.896	0.871	2.78 ± 1.02
ET6 How often do you experience an excellent organizational culture?	0.823	0.942	2.66 ± 1.128
Feedback and Organizational Support		0.793	3.31 ± 1.07
FOS2 How often do you receive verbal reviews from the evaluator regarding your performance?	0.806	0.611	3.48 ± 1.12
FOS5 How often do you receive completed feedback regarding your performance?	0.884	0.722	3.48 ± 1.26
FOS6 How often do you receive supervision?	0.841	0.596	3.21 ± 1.41
Incentives and Consequences		0.838	2.84 ± 1.16
IC2 How often have you received non-monetary incentives from your employer?	0.932	0.722	2.81 ± 1.28
IC3 How often have you received disincentives for poor performance?	0.924	0.722	2.86 ± 1.22
Health Status		0.800	2.94 ± 0.91
HS2 How often do you experience limitations in your daily activities?	0.862	0.737	4.17 ± 0.81
HS4 How often do you frequently encounter pain?	0.812	0.715	4.14 ± 0.79
HS5 How often do you experience limitations in social activities?	0.820	0.730	3.89 ± 1.08
Work-Family Conflict		0.792	3.08 ± 1.04
WFC1 How often do the demands of your family interfere with work-related activities?	0.914	0.656	3.16 ± 1.12
WFC2 How often do you have to put off doing things at work because of demands on your time at home?	0.906	0.656	2.99 ± 1.11
Healthcare provider performance		0.801	4.05 ± 0.79
HCP2 How often did you keep in mind the work result you needed to achieve?	0.817	0.635	4.17 ± 0.81
HCP3 How often were you able to set priorities?	0.845	0.660	4.09 ± 0.89
HCP5 How often did you manage your time well?	0.883	0.673	3.89 ± 1.08
Total		0.837	3.40 ± 0.59

### 3.3 Confirmatory factor analysis

**Figure 2:**
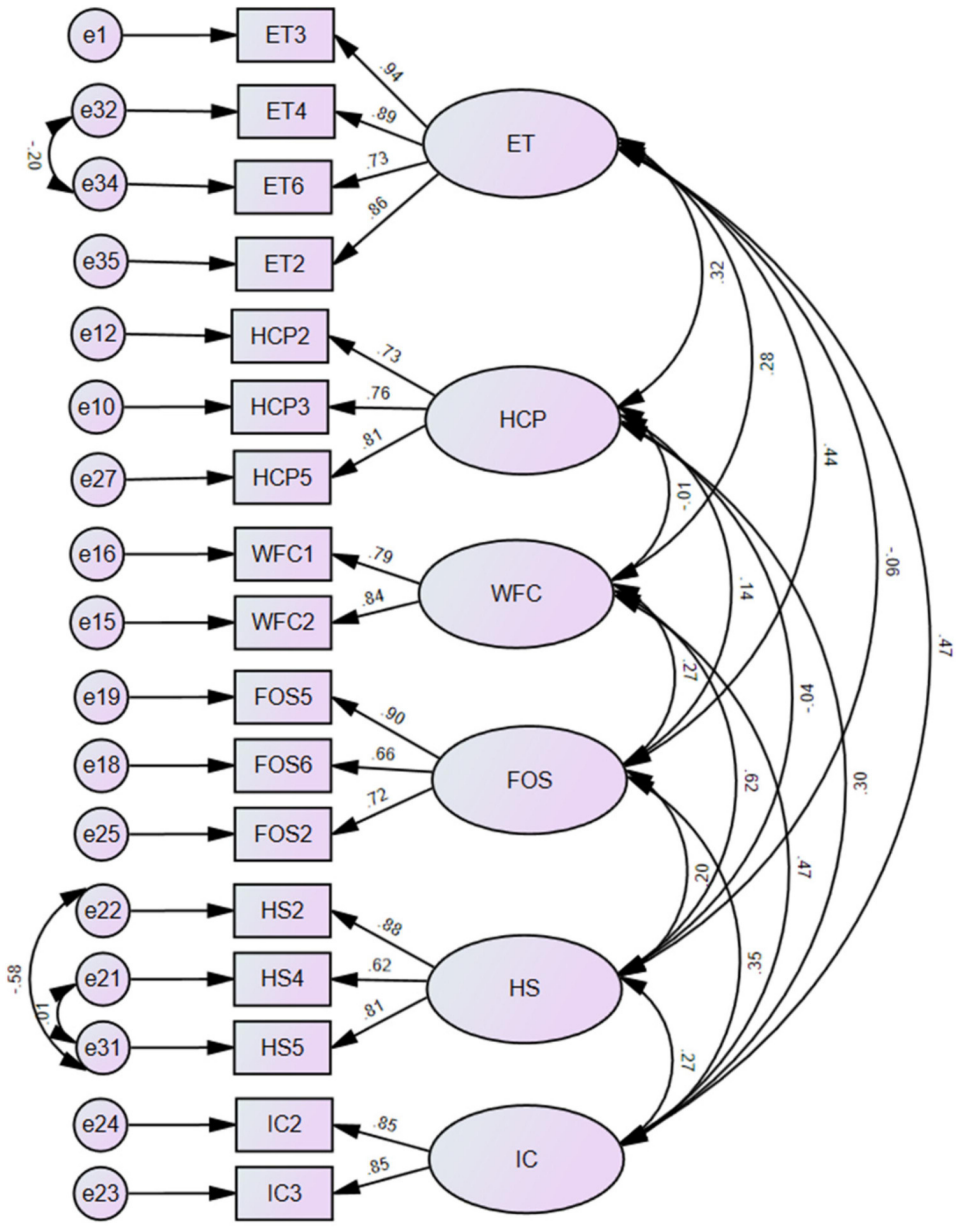
Confirmatory factor analysis diagram.

Figure 2 illsutrates the final CFA measurement model, which shows significant standardized factor loadings above 0.60 for all items. Nonetheless, inter-factor correlations support the distinctiveness and coherence of the latent constructs, and in order to enhance model fit, limited correlated error terms within constructs were used.

Given that *χ*² is highly sensitive to large sample sizes, alternative fit indices were examined to provide a balanced and accurate evaluation of the model fit. As presented in Table 3, the measurement of the six latent constructs (ET, HCP, WFC, HS, FOS, and IC) evaluated and provide an acceptable model fit values GFI = .91, AGFI = .86, CFI = .91, and NFI = .90. However, RMSEA 0.08 was slightly above the recommended cut off, which indicate moderate fit of the model. In addition to RMR 0.06, which is in the acceptable range.

**Table 3:** Indices of the confirmatory factor analysis.

Model fit indictors	Good fit	Acceptable fit	Results
RMSEA	≤ 0.05	≤ 0.08	0.08
RMR	≤ 0.05	≤ 0.08	0.06
CFI	≥ 0.95	≥ 0.90	0.91
GFI	≥ 0.95	≥ 0.90	0.91
AGFI	≥ 0.95	≥ 0.85	0.86
NFI	≥ 0.95	≥ 0.90	0.90

AGFI, adjusted goodness-of-fit index; CFI, comparative fit index; GFI, goodness-of-fit index; NFI, normed fit index; RMSEA, root mean square error of approximation; RMR, root mean square residual.

Table 4 illustrates the assessment of convergent and discriminant validity for the scale construct. Convergent validity was supported by AVE, which showed a considerably acceptable level greater than 0.50, indicating that the constructs explain more than half of the variance in the items. Discriminant Validity was also supported, in which AVE for each construct exceeded MSV and ASV.

**Table 4:** Convergent and discriminant validities assessment.

Construct	AVE	C.R	MSV	ASV
ET	0.737	0.917	0.243	0.131
WFC	0.665	0.799	0.406	0.160
FOS	0.588	0.808	0.210	0.109
HS	0.605	0.818	0.406	0.114
IC	0.723	0.839	0.243	0.164
HCP	0.589	0.811	0.109	0.048

AVE, average variance extracted; MSV, maximum shared variance; ASV, average shared squared variance; C.R, composite reliability; HCP, healthcare provider performance; FOS, feedback and organization support; ET, environment and tools; IC, incentives and consequences; HS, health status; WFC, work-family conflict.

Regarding discriminant validity, which was supported by both MSV and ASV, was reported lower than AVE for each construct in this model. The majority of constructs in this study satisfied this condition, indicating acceptable discriminant validity, such as WFC and HS, which showed MSV values (0.406) that are less than the AVEs (0.665 and 0.605, respectively). The overall results showed that the model has acceptable levels of convergent and discriminant validity for all constructs.

## 4 Discussion

The confirmed six-factor structure of the HCPP scale provides an adequate fit for the data, with six identified domains (Environment and Tools, Healthcare Provider Performance, Work-Family Conflict, Feedback and Organizational Support, Health Status, and Incentives and Consequences), which is consistent with previous research emphasizing the multifaceted nature of HCPP, where both individual and organizational factors can have an impact (55, 56).

Regarding the internal consistency of domains, which reflected an acceptable to excellent level of Cronbach's alpha. For instance, the environment and tools demonstrate the importance of adequate resources and a supportive organizational culture for HCPP. These findings align with previous investigations reporting that the strong reliability of workplace environment measures (57, 58). However, despite the high level of reliability, participants showed concern about the level of access to essential equipment and supplies, reflecting major challenges documented in the healthcare sector; these actual conditions may hinder optimal performance (59).

Incentives and Consequences and Feedback and Organizational domains showed good reliability. Participants illustrate the role of Incentives as an important component in terms of low receipt of non-monetary incentives, which is consistent with a previous study in similar healthcare settings where organizational support was recognized as insufficient, which could impact the performance (60). Notably, the Incentives and Consequences domain consisted of only two items, despite the low number of items for this factor, as recommended in the literature, two items can be identified when the error terms are uncorrelated (61). Furthermore, Feedback and supervision are considered important determinants of the HCPP. For instance, previous studies showed that supervision is important for organizations to develop and improve performance (62).

Regarding the Health Status domain, which revealed notable health challenges among healthcare providers, in terms of psychological, pain, and physical activity. Studies and findings from research on healthcare worker wellbeing, where musculoskeletal complications and frequent pain are prevalent, can impact performance (63). Nonetheless, the work-family conflict highlights the ongoing struggle to balance between the professional and family demands; these challenges are consistent with previous studies reported in other healthcare workforce studies (64). Despite these challenges with the factors associated with HCPP, a degree of self-efficacy and commitment can be observed in the context of healthcare, which concurs with investigations showing that healthcare providers want to maintain a high level of performance even under suboptimal conditions (65).

This study of the HCPP scale was rigorously developed and validated using a large and diverse sample of healthcare providers across various settings and locations to enhance the generalizability of findings. This scale captures the essence of the range of both individual and organizational factors influencing HCPP. Despite the strengths of the study, certain limitations were observed. Incentives and Consequences include only two items, which could limit the psychometric robustness. Future studies should expand the Incentives and Consequences domain by incorporating additional items to enhance reliability and content coverage. Nonetheless, testing the HCPP instrument in diverse healthcare systems and different cultural contexts to improve and strengthen its international applicability and cross-cultural validity. This validated HCPP tool provides policymakers and healthcare administrations with a practical tool for diagnosing the performance factors within the healthcare sector. By identifying the modifiable factors and determining the need to improve and maintain the level of performance among the workforce of healthcare providers. Nonetheless, this tool provides valuable insight into insufficient feedback, work-family conflict levels, and helps in providing targeted interventions to improve work conditions and well-being. Moreover, integrating the HCPP assessment into routine human resource evaluations can support strategic workforce planning and organizational development.

## 5 Conclusion

The validated and reliable HCPP scale demonstrated strong psychometric properties in assessing the factors associated with the healthcare provider performance. The six-factor structure (Feedback and Organizational Support, Environment and Tools, Incentives and Consequences, Health Status, Work-Family Conflict, and Healthcare Provider Performance) domains reflect the complexity of factors affecting providers in healthcare settings. This scale at hand provides a valuable instrument for future research and practical interventions aimed at improving the healthcare provider performance nationally and globally. Nonetheless, the health sector and the general management can use this scale to assess performance, guide workforce development, and implement targeted interventions, ultimately improving the quality of services and enhancing organizational effectiveness.

## Data Availability

The raw data supporting the conclusions of this article will be made available by the authors, without undue reservation.
