# Supplementary material for: Construct validity and internal reliability of the healthcare provider performance scale
Source: Front Health Serv. 2026 Feb 9;6:1735784. doi: 10.3389/frhs.2026.1735784 (PMC12926396; doi:10.3389/frhs.2026.1735784)
Supplement: Supplementary file 1 [file Table1.docx]

**Healthcare Provider Performance (HCPP) Questions**

**Healthcare Provider Performance**

|  | **Statement** |
| --- | --- |
| HCP1 | How often were you able to plan your work so that you finished it on time? |
| HCP2 | How often did you keep in mind the work result you needed to achieve? |
| HCP3 | How often were you able to set priorities? |
| HCP4 | How often were you able to carry out your work efficiently? |
| HCP5 | How often did you manage your time well? |
| HCP6 | How often did you, on your own initiative, start new tasks when your old tasks were completed? |
| HCP7 | How often did you come up with creative solutions for new problems? |
| HCP8 | How often did you take on extra responsibilities? |
| HCP9 | How often did you continually seek new challenges in your work? |

**Feedback and Organizational Support**

|  | **Statement** |
| --- | --- |
| FOS1 | How often do you receive verbal reviews from the evaluator regarding your performance? |
| FOS2 | How often do you receive written reviews from the evaluator regarding your performance? |
| FOS3 | How often do you receive feedback from the evaluator regarding your performance? |
| FOS4 | How often do you receive completed feedback regarding your performance? |
| FOS5 | How often do you receive supervision during a six-month period? |
| FOS6 | How often have you been engaged to perform work by your organization? |
| FOS7 | How often does your organization ignore your need to perform the job to the best of your ability? |
| FOS8 | How often does your organization provide orientation for their employees? |

**Knowledge and Skills**

|  | **Statement** |
| --- | --- |
| KS1 | How often have you received training in a health-related field? |
| KS2 | How often have you received training in the use of healthcare tools? |
| KS3 | How often do you feel you have the necessary knowledge to perform your job? |
| KS4 | How often do you feel you have the necessary skills to perform your job? |

**Clear Job Expectations**

|  | **Statement** |
| --- | --- |
| CJE1 | How often are you aware of and acknowledge your job description? |
| CJE2 | How often are you involved in discussing your roles and tasks? |
| CJE3 | How often have your performance measurements been objectively set? |

**Environment and Tools**

|  | **Statement** |
| --- | --- |
| ET1 | How often do you find your workplace to be adequate? |
| ET2 | How often do you have the necessary equipment to perform your job? |
| ET3 | How often are you satisfied with the work regulations? |
| ET4 | How often do you have the necessary instruments to perform your job? |
| ET5 | How often do you have the necessary supplies to perform your job? |
| ET6 | How often do you experience an excellent organizational culture? |

**Incentives and Consequences**

|  | **Statement** |
| --- | --- |
| IC1 | How often do you have opportunities for promotion? |
| IC2 | How often have you received non-monetary incentives from the employer? |
| IC3 | How often have you received disincentives for poor performance? |
| IC4 | How often have you received bonuses or raises for good performance? |
| IC5 | How often have you received non-monetary incentives from the community? |

**Health Status**

|  | **Statement** |
| --- | --- |
| HS1 | In general, how would you rate your health? |
| HS2 | How often do you experience limitations in your daily activities? |
| HS3 | How often do you experience limitations in walking uphill or climbing stairs? |
| HS4 | How often do you frequently encounter pain? |
| HS5 | How often do you experience limitations in social activities? |
| HS6 | How often have you felt calm and peaceful during the past month? |

**Work-Family Conflict**

|  | **Statement** |
| --- | --- |
| WFC1 | How often do the demands of your family interfere with work-related activities? |
| WFC2 | How often do you have to put off doing things at work because of demands on your time at home? |
| WFC3 | How often do family-related matters interfere with your ability to perform job-related duties? |
